# Advances in Treatment of Dyslipidemia

**DOI:** 10.3390/ijms241713288

**Published:** 2023-08-27

**Authors:** Jill Dybiec, Wiktoria Baran, Bartłomiej Dąbek, Piotr Fularski, Ewelina Młynarska, Ewa Radzioch, Jacek Rysz, Beata Franczyk

**Affiliations:** 1 Department of Nephrocardiology, Medical University of Lodz, ul. Zeromskiego 113, 90-549 Lodz, Poland; 2 Department of Nephrology, Hypertension and Family Medicine, Medical University of Lodz, ul. Zeromskiego 113, 90-549 Lodz, Poland

**Keywords:** dyslipidemia, cholesterol, cardiovascular disease, familial hypercholesterolemia, alirocumab, lomitapide

## Abstract

Dyslipidemias have emerged as prevalent disorders among patients, posing significant risks for the development and progression of cardiovascular diseases. These conditions are characterized by elevated levels of total cholesterol (TC), triglycerides (TGs), and low-density lipoprotein cholesterol (LDL-C). This review delves into the current treatment approach, focusing on equalizing these parameters while enhancing the overall quality of life for patients. Through an extensive analysis of clinical trials, we identify disorders that necessitate alternative treatment strategies, notably familial hypercholesterolemia. The primary objective of this review is to consolidate existing information concerning drugs with the potential to revolutionize dyslipidemia management significantly. Among these promising pharmaceuticals, we highlight alirocumab, bempedoic acid, antisense oligonucleotides, angiopoietin-like protein inhibitors, apolipoprotein C-III (APOC3) inhibitors, lomitapide, and cholesterol ester transfer protein (CETP) inhibitors. Our review demonstrates the pivotal roles played by each of these drugs in targeting specific parameters of lipid metabolism. We outline the future landscape of dyslipidemia treatment, envisaging a more tailored and effective therapeutic approach to address this widespread medical concern.

## 1. Introduction

Dyslipidemias can be characterized by an elevated level of TC (total cholesterol), LDL-C (low-density lipoprotein cholesterol), TGs (triglycerides), a lowered level of HDL-C (high-density lipoprotein cholesterol) within the blood plasma, or by blends of the mentioned elements. In other words, they constitute metabolic disturbances associated with the lipid profile. Dyslipidemia prevalence has increased over the last several years, and it often happens to be the starting point of cardiovascular disease [1]. This lipid alteration has various etiologies that can be divided into primary and secondary. The first group consists of genetic diseases, such as familial chylomicronemia syndrome (FCS), familial dysbetalipoproteinemia (FD), familial hypertriglyceridemia (FHTG), homozygous familial hypercholesterolemia (HoFH), autosomal recessive hypercholesterolemia (ARH), et cetera, while the second group includes functioning disorders of specific organs, namely the kidneys, thyroid gland, and liver, or disorders that may occur due to taking certain medications like steroids or selected beta-adrenergic blockers [2]. What is more, a sedentary lifestyle and unhealthy diet also have their own role in dyslipidemia development, even among the youth [3]. There is also a term known as “diabetic dyslipidemia”, which refers to elevated levels of TGs, TRLs (triglyceride-rich lipoproteins), and LDL-C while the level of HDL is lowered. This specific profile highly increases the risk of cardiovascular events and, due to that, requires appropriate attention [4]. However, thanks to specific medications, nutraceuticals, and exercising, we have the capability to affect dyslipidemia, which allows for the reduction in cardiovascular risk in this group of patients [4,5,6]. In this review, we are especially focused on discussing the topic of the latest pharmacological treatment of the mentioned disorder. Nowadays, there are few groups of medicines used for dyslipidemia treatment. Besides statins, ezetimibe, or a combination of statins with ezetimibe, for this purpose, there is the possibility to use monoclonal antibodies binding with PCSK 9 (proprotein convertase subtilisin/kexin type 9), such as alirocumab or inhibitors of ATP (adenosine triphosphate) citrate lyase, like bempedoic acid. Moreover, when it comes down to normalizing lipid levels, antisense oligonucleotides, angiopoietin-like protein inhibitors, cholesteryl ester transfer protein inhibitors (CETPis), microsomal triglyceride transfer protein (MTP) inhibitors such as lomitapide, or apolipoprotein C-III (APOC3) inhibitors play an important role. Furthermore, there is also an older generation of drugs available, namely bile acid-binding agents, such as colesevelam and cholestyramine, that can also reduce LDL-C levels in the bloodstream [7,8,9].

## 2. Epidemiology

Dyslipidemia is a medical condition characterized by abnormal concentrations of lipids and lipoproteins in the plasma, deviating from values considered desirable. Numerous studies have indisputably demonstrated that hyperlipidemia is the leading global cause of morbidity and mortality, serving as the primary etiological factor for cardiovascular disease (CVD) and its associated condition, atherosclerotic cardiovascular disease (ASCVD) [10,11,12].

A considerable proportion of hyperlipidemic patients suffer from a primary lipid disorder. Additionally, secondary hyperlipidemia can arise as a result of various conditions, including diabetes mellitus, obesity, excessive alcohol consumption, hypothyroidism, kidney disease (e.g., chronic renal failure), liver disease (e.g., cholestasis), and certain medications (e.g., thiazide diuretics, beta blockers, or atypical antipsychotics) [13]. One serious type of primary dyslipidemia is familial hyperlipidemia (FH), which can be attributed to genetic mutations affecting critical lipid metabolism genes, leading to significantly elevated levels of LDL-C. This condition is often accompanied by a strong family history of premature CVD and the occurrence of cardiovascular events at relatively young ages [14,15].

Due to the pivotal role of lipids and lipoproteins in CVD, they have become the subject of numerous studies. For instance, significant differences in cholesterol concentrations have been observed between Caucasians and Black people. Black people tend to have lower mean serum TC and triglyceride, or TG, levels, higher HDL-C, and a lower prevalence of hypercholesterolemia and hypertriglyceridemia. Conversely, White people exhibit higher mean serum TC and TG levels, lower serum HDL-C, and a higher prevalence of hypercholesterolemia and hypertriglyceridemia. Furthermore, women aged 65 and older have significantly higher TC and TG levels than men in the same age group. Smokers also have higher overall mean TC, TG, and LDL-C levels compared to non-smokers [16].

In 2020, approximately 4.51 million deaths were attributed to high levels of LDL-C, marking a 19% increase in deaths compared to 2010. According to data from the American Heart Association (AHA), between 2017 and 2020, approximately 32.8% of US adult males and 36.2% of US adult females had TC levels ≥ 5.172 mmol/L (200 mg/dL). A level of LDL-C ≥ 3.362 mmol/L (130 mg/dL) was observed in 25.6% of United States (US) males and 25.4% of US females. Additionally, 24.9% of US males and 9.3% of US females reported HDL-C levels < 1.034 mmol/L (40 mg/dL) [17].

Data analysis of lipid panels from 2017 to 2020 for US adults yielded the following results: the average TC level was 4.84 mmol/L (187.2 mg/dL), the average level of LDL-C was 2.847 mmol/L (110.1 mg/dL), the average level of HDL-C was 1.386 mmol/L (53.6 mg/dL), and the average TG level was 1.034 mmol/L (91.6 mg/dL) [17].

An ESC (European Society of Cardiology) data analysis for 2018 showed the following results: the mean TC level was 4.82 mmol/L (186.4 mg/dL) in males and 4.92 mmol/L (190.26 mg/dl) in females. The median non-HDL-C level was 3.36 mmol/L (129.93 mg/dL) for females and 3.53 mmol/L (136.5 mg/dL) for males. The median HDL-C level was 1.53 mmol/L (59.16 mg/dL) for females and 1.27 mmol/L 49.11 mg/dL) for males. The LDL-C parameter was not mentioned [18].

## 3. Primary Prevention and Target Lipid Values

Guidelines highlight the importance of a healthy diet, such as sodium restriction, reducing the intake of saturated fatty acids, moderating alcohol consumption, and adopting proper lifestyle habits, in the primary prevention of dyslipidemia [19]. It is recommended to increase physical activity through regular exercise and avoid a sedentary lifestyle [20,21]. If the goal is not achieved or the patient belongs to a different risk group than low risk for CVD, then, in addition to diet and lifestyle changes, pharmacological treatment is recommended.

**Figure 1:**
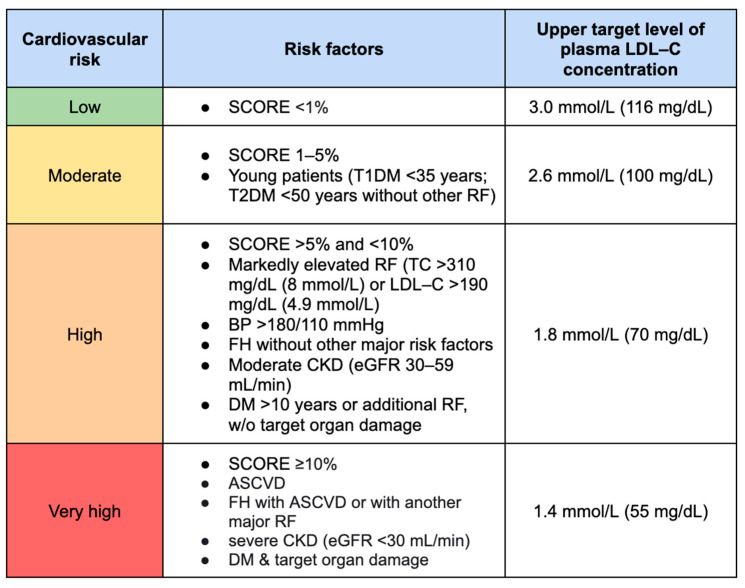
Therapeutic goals for low-density lipoprotein cholesterol depending on cardiovascular risk. ASCVD, atherosclerotic cardiovascular disease; BP, blood pressure; CKD, chronic kidney disease; eGFR, estimated glomerular filtration rate; FH, familiar hypercholesterolemia; LDL-C, low-density lipoprotein cholesterol; RF, risk factor; SCORE, Systematic Coronary Risk Evaluation; T1DM, type 1 diabetes mellitus; T2DM, type 2 diabetes mellitus; TC, total cholesterol.

The main goal of prevention and subsequent therapy is to lower the LDL-C value in order to reduce the risk of cardiovascular death across all risk groups. The categorization of patients into specific cardiovascular risk groups depends, among other factors, on the presence of comorbidities (Figure 1).

Recent studies recommend reducing LDL-C by ≥50% compared to the baseline value and aiming for a target level of <55 mg/dL in the very-high-risk group. Moreover, it is suggested to maintain LDL-C levels at <1.8 mmol/L (<70 mg/dL) in high-risk groups, <2.6 mmol/L (<100 mg/dL) in moderate-risk groups, and <3.0 mmol/L (<116 mg/dL) in low-risk groups [20].

According to ESC guidelines, the goal for non-HDL-C levels is <2.2, 2.6, and 3.4 mmol/L (<85, 100, and 130 mg/dL) for very-high-, high-, and moderate-risk people [20]. There is no target value of TGs, but a TG level < 1.7 mmol/L (<150 mg/dL) indicates a lower CV risk. In patients with a high CVD risk and elevated fasting TG level (1.7 mmol/L and more), and who are taking statins, NICE guidelines recommend icosapent ethyl (Vazkepa) as a drug opportunity for reducing CVD risk in adults. This medication is recommended for patients who have established CV disease and LDL-C levels above 1.04 mmol/L and below or equal to 2.60 mmol/L (secondary prevention), or diabetes and at least one other CV risk factor (primary prevention). Statin intake is required to implement Vazkepa [22].

## 4. Screening for Dyslipidemia

Recommendations for screening adults for dyslipidemia are unclear. It is suggested that there are no indications for screening patients with risk factors until at least 40 years of age [23]. The Canadian Cardiovascular Society (CCS) recommendations propose testing the lipid profile in individuals over 40 years of age or those with risk factors, regardless of gender [20]. On the other hand, other guidelines advocate for lipid profile monitoring starting at the age of 20. In this scenario, retesting should be performed between 25 and 30 years of age for men and between 30 and 35 years of age for women, especially if they belong to the high-risk group [24]. If the risk is lower, the next lipid profile test should be conducted at the age of 35 or 45 for men and women, respectively [25]. The earlier initiation of monitoring is supported by the possibility of earlier dyslipidemia diagnosis, leading to prompt intervention and the prevention or deceleration of cardiovascular complications primarily caused by elevated LDL-C levels.

### Screening Tests

The screening method most frequently suggested by guidelines is the standard lipid profile. It includes TC, HDL-C, non-HDL-C, LDL-C, and TGs [24,26]. Some guidelines, in addition to the standard lipid profile, mention the measurement of ApoB-100, as this parameter considers all lipoproteins that are deemed atherogenic [25]. It is crucial to note that LDL, VLDL (very-low-density lipoprotein), and TGs are correlated with an increased risk of coronary heart disease (CHD), whereas HDL has the opposite effect, and higher levels of HDL may exert a protective effect on CHD and mortality.

The measurement of LDL-C is recommended as the primary parameter for lipid analysis, and in the case of screening tests, determining the lipid profile in a non-fasting state is preferred [20]. Estimating the LDL level can be achieved through indirect and direct methods. The indirect method involves calculating the LDL-C level using the Friedewald formula, which takes into account total cholesterol, VLDL-C, and HDL-C. VLDL-C is estimated by dividing the measured TG level by 2.2 in mmol/L or 5 in mg/dL. However, in cases where the TG level > 4.5 mmol/L (>400 mg/dL), the calculation of LDL-C using the formula is invalid, leading to an incorrect determination of its actual level [27]. Furthermore, in patients with elevated TG levels, non-fasting measurements may result in miscalculated LDL-C levels. For patients with a TG concentration > 4.5 mmol/L (>400 mg/dL), it is recommended to perform the test in a fasting state [20] or to consider direct LDL measurement. Nonetheless, the direct measurement method is subject to certain errors, which means that the values obtained may differ from the calculated values.

When calculating the LDL value, it should be taken into account that patients with obesity or type 2 diabetes usually have an overproduction and secretion of atherogenic VLDL. Moreover, patients with insulin resistance commonly have a higher burden of small, dense LDL (sdLDL) and a reduced high-density lipoprotein (HDL) production [28]. An elevated LDL level correlates with an increased CVD risk, independently of other lipid markers. Thus, it should be considered during serum LDL measurement and when calculating the risk of atherogenic CVD.

Non-HDL-C is calculated by subtracting the measured HDL-C from TC. Due to its inclusion of the cholesterol present in all atherogenic lipoproteins, it can more accurately determine the risk of atherogenicity compared to LDL-C. Moreover, ApoB may provide information on the quantity of atherogenic lipoproteins [23,29] and potentially offer a better risk assessment, particularly in individuals with insulin resistance. The guidelines from the ESC recommend measuring non-HDL-C and ApoB in all patients with high TG levels, diabetes, and obesity, while the American Heart Association/American College of Cardiology/Multisociety guidelines [24] suggest not routinely measuring ApoB, considering cost-effectiveness issues, and recommend performing this test when TG levels ≥ 200 mg/dL [20].

When initiating treatment for dyslipidemia, it should be taken into account that the lipid profile may be less accurate in the presence of high concentrations of abnormal monoclonal proteins, as observed in post-acute coronary syndrome (ACS) or post-surgical conditions [20].

The Lp(a) level serves as a genetic risk factor for ASCVD. Therefore, the guidelines recommend measuring this indicator at least once in a patient’s lifetime, especially if they have a family history of premature ASCVD. It is worth mentioning that there are many controversies related to the determination of Lp(a), because despite many studies, its definitive utility in the diagnosis of dyslipidemia remains unsubstantiated [30]. This situation may change, because in recent years, studies on a new drug (olpasiran) have been conducted, which have proven that the therapy significantly reduces Lp(a) levels in patients with established atherosclerotic cardiovascular disease [31]. Further comprehensive investigations into olpasiran and its implications on CVD are needed. It is plausible that the continued research of this drug may facilitate the establishment of Lp(a) as a screening parameter for dyslipidemia diagnosis.

The NICE guidelines mention the use of cascade testing among relatives of patients diagnosed with FH as a screening modality for these individuals. This methodology, aimed at identifying biological relatives at risk of a genetic disease, entails the performance of a DNA test in cases where a disease-causing mutation has been identified in the index individual or proband [32]. The guidelines pertaining to genetic testing in dyslipidemias emphasize the significance of DNA testing for suspected FH, FCS, and rare monogenic dyslipidemias [33]. The advent of genetic diagnostic tests has enabled the unequivocal diagnosis of dyslipidemia, enhanced prognosis determination, and facilitated targeted treatment implementation. Genetic tests conducted on family members expedite disease diagnosis, permit earlier intervention, and facilitate the implementation of appropriate treatment strategies. Genetic diagnostics should be considered when the laboratory parameter LDL-C is >5 mmol/L (>194 mg/dL), while for FCS, when TG levels > 10 mmol/L (<885 mg/dL), in patients devoid of secondary causes. The cascade screening tests may also be performed on children. A family history indicative of a lipid phenotype or early ASCVD should prompt contemplation of such screening methods [25].

## 5. New Drugs in Treatment of Dyslipidemia

Conventional lipid-lowering therapies (LLTs), statins, ezetimibe, and PCSK9-inhibitors, coupled with maintaining a healthy lifestyle, are the foundation of treatment to reduce cholesterol levels. Statins limit cholesterol biosynthesis by decreasing the cellular cholesterol content by selectively inhibiting the enzyme HMG-CoA reductase [34]. Meta-analysis of 62 trials showed that statin treatment may have side effects, like muscle problems (myalgia, myopathy, or rhabdomyolysis), liver and renal dysfunction, diabetes mellitus type 2, or eye conditions like cataracts, that limit the achievement of safe LDL-C levels and may be a reason for the discontinuation of the therapy [35]. Ezetimibe stands as the primary option for pairing with the maximum tolerated statin dose if the LDL-C objective is not met. Ezetimibe operates by disrupting the absorption of cholesterol in the intestines, and its effectiveness hinges on the presence of the Niemann-Pick1-like protein [36]. Combined statin–ezetimibe therapy led to further reductions in LDL cholesterol levels and enhanced cardiovascular outcomes [37]. However, the Getting to an Improved Understanding of Low-Density Lipoprotein Cholesterol and Dyslipidemia Management (GOULD) study showed that only 21% (LDL-C baseline: (2.6 mmol/L) 100 mg/dL and more) and 33.9% (LDL-C baseline: 1.8–2.56 mmol/L (70 to 99 mg/dL)) of patients with conventional LLTs achieved an LDL-C less than 1.8 mmol/L (70 mg/dl) at 2 years. What is more, only 10% (LDL-C baseline: 2.6 mmol/L (100 mg/dL) or more) and 11.9% (LDL-C baseline: 1.8–2.56 mmol/L (70 to 99 mg/dL)) of patients with conventional LLTs achieved an LDL-C level of less than 55 mg/dL at 2 years [38]. In addition to side effects after using conventional LLTs, LLTs also have other limitations in achieving safe cholesterol levels. Numerous factors hinder the effectiveness of traditional LLTs. These include issues such as genetic factors and decreased production of LDL receptors [39,40,41], the enhanced synthesis of cholesterol due to ezetimibe [42] or metabolic processes impacting the transformation of VLDL to LDL, and slight lipoprotein lipase (LPL) activity [43,44]. Therefore, it is crucial to deepen the knowledge about lipid metabolism, which would allow the finding of crucial components that may be the point of action of new LLTs. For such, new drugs can be considered: alirocumab, bempedoic acid, ASOs, ANGPTL inhibitors, APOC3 inhibitors, lomitapide, and CETP inhibitors [15,20]. Bempedoic acid operates in the same metabolic pathway as statins, but at an earlier stage, and acts by inhibiting ATP citrate lyase (ACLY). Furthermore, therapies centered around proprotein convertase subtilisin-kexin 9 (PCSK9) adjust this pathway by preventing the degradation of LDLR in lysosomes. Inclisiran, a small interfering RNA directed at PCSK9 messenger RNA (mRNA), attaches to the asialoglycoprotein receptor (ASGPR) to achieve a similar effect. APOC3 inhibitors and ANGPTL3 inhibitors have alternative points of action, and both inhibit the LPL pathway. Lomitapide works by directly binding to MTP, hindering lipid transfer, and blocking its function in both the liver and intestines [8,36].

### 5.1. Alirocumab

Alirocumab (Praluent) is a human immunoglobulin G1 monoclonal antibody derived from VelocImmune mice, in which both light and heavy immunoglobulin chains have been replaced with human equivalents [45,46]. This drug was developed by Regeneron Pharmaceuticals and Sanofi and has been approved by the US [47,48]. The mechanism of this drug is to act against the proprotein convertase subtilisin/kexin type 9(PCSK9) [49,50]. As a result, there is an increase in the hepatic uptake of LDL-C by the binding of the drug to receptors located in hepatocytes [51]. The effect reduces the concentration of not only LDL-C, but also non-HDL-C, apoB, apolipoprotein A, and lipoprotein A. The drug is administered subcutaneously at a dose of 75–150 mg every 2 weeks [45]. Its indication is for patients taking maximum doses of statins with clinical ASCVD or patients with familial hypercholesterolemia (FH) [48]. There are also papers suggesting a positive cardiovascular effect in post-acute coronary syndrome patients using statins [52]. Alirocumab has a fairly high tolerability profile [50]. However, there have been reported instances of side effects in clinical trials, as depicted in Table 1 [48].

**Figure 2:**
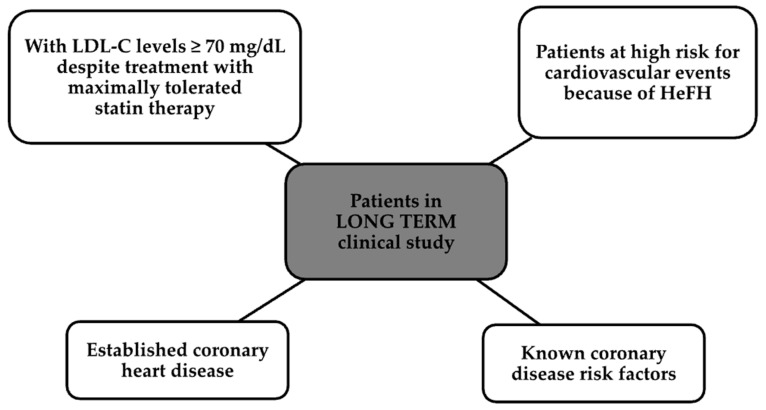
Patient groups participating in the clinical trial.

One randomized long-term study was double-blind and included more than 2300 patients, whose groups are shown in Figure 2 [51].

Patients were given alirocumab 150 mg subcutaneously or a placebo every 2 weeks for 78 weeks. The administration of this drug resulted in a decrease in LDL-C by as much as 61% after 24 weeks and over 52% after more than 78 weeks [51,53].

In another randomized double-blind study, HIGH FH, 107 patients taking the maximum tolerated dose of statins had LDL-C levels of 160 mg/dL. After receiving alirocumab at a dose of 150 mg subcutaneously every 2 weeks, LDL-C levels dropped by 43% after 24 weeks [51]. Another two randomized trials on 660 patients showed that using alirocumab at a dose half that of the other studies, i.e., 75 mg, resulted in a greater reduction in LDL-C than switching from atorvastatin to rosuvastatin, adding ezetimibe, or increasing the current statin dose by half [51,54].

### 5.2. Evolocumab

Another human monoclonal G2 antibody belonging to PCSK9 is Evolocumab, known by trade names like Repatha^®^ or Amgen [55,56]. Its mode of action is to increase LDL-C uptake by blocking the binding of PCSK9 to the LDL receptor [57]. This drug is administered at a dose of 140 mg subcutaneously every 2 weeks or once a month at a dose of 420 mg. The indication for evolocumab is mixed dyslipidemia, primary hypercholesterolemia, and homozygous familial hypercholesterolemia, especially concerning patients who do not respond well to treatment with statins or cannot take them [56]. In clinical trials, Evolocumab has been proven to reduce LDL-C levels by up to more than 50%, as well as lipoprotein a and other lipid levels, compared to the placebo. The half-life of this drug is up to 17 days, and the maximum serum concentration is reached after 3–4 days [55]. Recent studies also show that the use of this drug reduces cardiovascular risk and leads to a reduction in atherosclerotic plaque [57].

### 5.3. Bempedoic Acid

Bempedoic acid (8-hydroxy-2,2,14,14-tetramethylpentadecanoic acid) is known as NEXLETOL^®^ in the United States, or in the European Union, being developed by Esperion Therapeutics, as Nilemdo^®^ [58,59]. It was approved by the FDA (Food and Drug Administration) in February 2020. It is primarily indicated for adults with HeFH (heterozygous familial hypercholesterolemia) or established ASCVD. It is worth mentioning that recently, the FDA also approved a new drug that combines bempedoic acid and ezetimibe (Nexletol^®^) in a mono tablet form and has exactly the same indications [60].

Bempedoic acid is a pro-drug that regulates LDL-C receptors and influences the lowering of this fraction in patients with hypercholesterolemia [59]. It acts by inhibiting ATP citrate lyase (ACLY); that is, it acts at an earlier site in the enzymatic pathway than statins, affecting the later enzyme, 3-hydroxy-3-methylglutaryl coenzyme A (HMG-CoA) reductase [58,61]. It is noteworthy that bempedoic acid is a pro-drug, and its action begins after activation by liver-derived acyl-CoA synthetase 1 [58]. Its half-life, however, ranges from 15 to 24 h [62]. This pro-drug is administered orally at a dose of 180 mg daily, before or with meals, and reaches its stable concentration within 7 days. More than 70% of the drug is excreted in the urine, while the remaining 30% is excreted in the feces [63]. Bempedoic acid has been demonstrated to be well tolerated and safe. The significant difference in its safety compared to statins is due to the fact that muscle tissue is not exposed to the active metabolite of the acid described [64].

**Figure 3:**
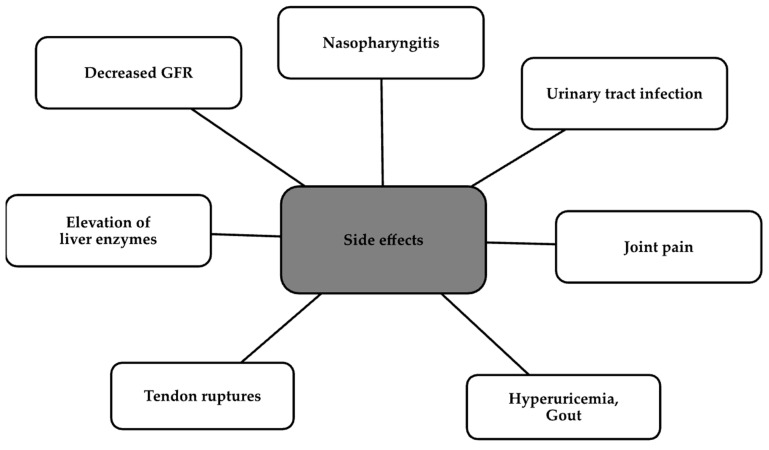
Side effects of bempedoic acid [67].

In preclinical studies, bempedoic acid has been proven to reduce triglycerides, glucose, or hs-CRP (high-sensitivity C Reactive Protein) levels, in addition to lowering LDL-C levels [62,65]. In animal models, it has also shown pleiotropic effects by reducing abdominal obesity, atherosclerotic plaques, or proinflammatory cytokine levels [62]. An important advantage of bempedoic acid is its ability to reduce inflammation, which shows a clear effect on reducing the risk of reinfarction in the JUPITER trial in patients taking concomitant rosuvastatin [66]. In contrast, four clinical trials evaluated its efficacy and safety; these were as follows: CLEAR Tranquility, CLEAR Serenity, CLEAR Harmony, and CLEAR Wisdom, which showed a percentage decrease in LDL-C levels compared to the placebo in all four trials. In these trials, most adverse events were considered mild or moderate. The most common events are depicted in Figure 3 [67].

Certainly, the long-term effects on the cardiovascular system will be evaluated in a growing number of studies to determine its long-term safety [67].

### 5.4. Antisense Oligonucleotides (ASOs)

**Figure 4:**
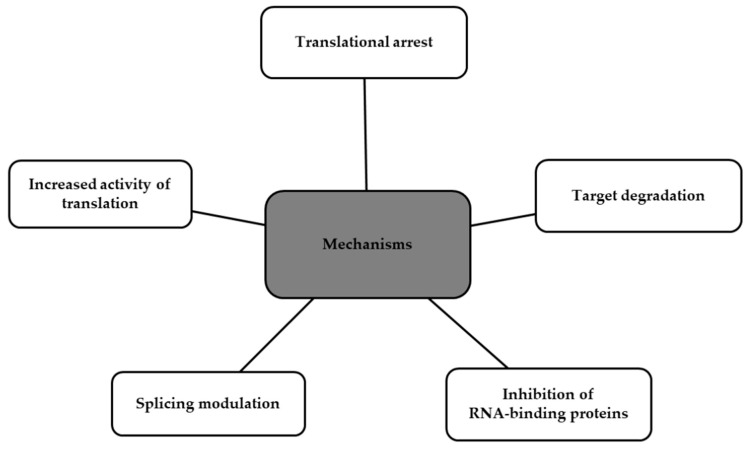
Functional mechanisms of ASOs.

Antisense oligonucleotides (ASOs) are synthetically manufactured, single-stranded, small molecules composed of modified DNA [68,69,70]. They bind complementary to target RNA (ribonucleic acid) via Watson–Crick hybridization, leading to the blockage of protein translation or inactivating genes responsible for disease [71,72]. Several mechanisms of ASOs can be distinguished, as shown in Figure 4 [69].

Importantly, ASOs are metabolized by cellular endonucleases and exonucleases, and not by the CYP450 (Cytochromes P450) hepatic system, which reduces the potential risk of drug interactions [73]. Clinical trials have been conducted using ASOs to reduce levels of lipoprotein(a). The first drug that specifically targeted apolipoprotein(a) m(messenger)RNA was an IONIS-APO(a)Rx-2′-O-methoxyethyl-modified ASO. Another modified version is Pelacarsen, which is a ligand-conjugated ASO with a triantennary GalNAc complex, which shows higher potency than the first one. In a phase 1 trial, six doses of IONIS-APO(a)Rx were administered, and the results were prominent. The reduction in Lp(a) levels was dose-related: for the 100 mg group, 39%; for the 200 mg group, 59%; and for the 300 mg group, 77%. In a phase 2 trial for the same drug, the results for Lp(a) level reduction were as follows: 67% in group A and 72% in group B. In a phase 1 trial of pelacarsen, it achieved an up to 92% Lp(a) reduction. Its phase 2 trial included participants with established ASCVD and Lp(a) levels > 60 mg/dL. The drug was administered in increasing doses at intervals of 1 to 4 weeks. Results after 6 months are presented in Table 2 [74,75,76].

A drug worth mentioning is Inclisiran (Leqvio^®^; Novartis, Basel, Switzerland). It is a cholesterol-lowering GalNAc-conjugated siRNA. Based on the results of the Orion trials, it was approved in December 2020. Its approval is for the treatment of adults with primary hypercholesterolemia (heterozygous familial and non-familial) or mixed dyslipidemia. Inclisiran has been shown to effectively reduce LDL-C levels in patients with ASCVD. It can be used alone or with other lipid-lowering therapies [77].

### 5.5. Angiopoietin-like Protein Inhibitors

**Figure 5:**
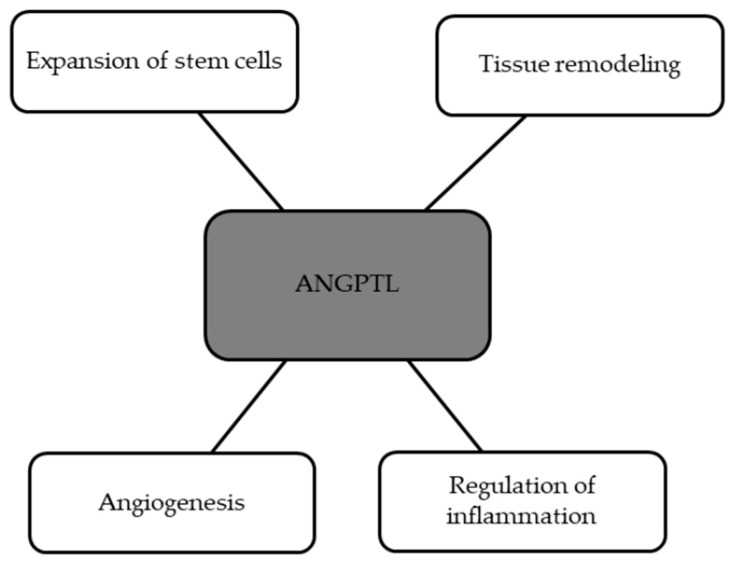
Processes with involvement of ANGPTL.

Until now, eight glycoproteins have been discovered that belong to the family of angiopoietin-like proteins (ANGPTL)—ANGPTL1–ANGPTL8. This family is a part of the vascular endothelial growth factor (VEGF) family and exhibits high homology to angiopoietins, which are suggested to take part in angiogenesis [78,79]. Figure 5 shows the processes in which ANGPTL has an involvement [78].

Among these proteins, ANGPTL3, ANGPTL4, and ANGPTL8 are the ones that regulate the activity of LPL in a coordinated way, and in consequence, regulate the lipolysis of TGs in TRLs [7,80,81]. ANGPTL3 and ANGPTL8 are synthesized and secreted by the liver, whereas ANGPLT4 mostly occurs in the adipose tissues. ANGPTL8 is an activator of ANGPTL3 that enhances its LPL (lipoprotein lipase) inhibitory actions in the heart and muscle [80,82]. It is worth noting that while fasting, ANGPLT4 expression is increased, ANGPLT8 expression is decreased, and ANGPLT3 expression remains the same. LPL activity decreases in adipose tissues but elevates in the heart and muscles. That leads to moving fatty acids and TGs away from the adipose tissue. On the other hand, while eating, ANGPTL4 expression decreases, and ANGPTL8 expression increases, leading to a renewal of LPL activity in adipose tissues to take up TGs for storage [78,80].

Evinacumab, distributed as EvkeezaTM, is a monoclonal antibody targeting ANGPTL3, which was developed by Regeneron Pharmaceuticals Inc. with the usage of Ve-locImmune technology [79,83]. Clinical trials have shown promising results in reducing TGs, LDL-C, and HDL-C [79,84,85]. Also, it appears that evinacumab may have an additive effect on the reduction in TG and LDL-C levels on top of PCSK9 inhibitors and statin treatment [85]. In a phase 3, placebo-controlled, randomized, double-blind trial, patients with HoFH were randomly assigned in a ratio of 2:1. One group, beside stable lipid-lowering therapy, received intravenous evinacumab every 4 weeks, while the second group was given a placebo. The percent change from baseline in LDL levels at week 24 was considered the primary outcome. In the beginning, the mean baseline LDL level in both groups was the same (255.1 mg per deciliter). At week 24, the group of patients that received evinacumab had a distinct reduction of 47,1% from baseline LDL levels, as compared to the placebo group, which showed an increase of 1,9% in the LDL levels. Adverse events were similar in both groups. The most common among them was nasopharyngitis [86].

### 5.6. APOC3 Inhibitors

**Figure 6:**
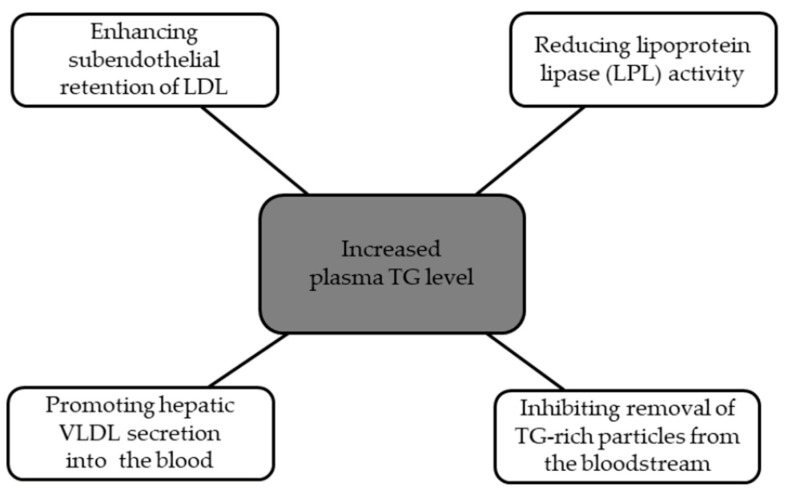
Mechanisms of apolipoprotein C3.

Apolipoprotein C3 (apoC3) is a glycoprotein that consists of 79 amino acids and contains two amphipathic helices [7,87]. It is mainly secreted from the liver and small intestine [7,88]. ApoC3 plays a crucial role in regulating TG metabolism [80]. It can be found in chylomicrons and remnant particles, and it is known to inhibit the activity of the lipoprotein lipase (LPL) and hepatic uptake of triglyceride-rich lipoproteins (TGRLs) [89,90]. It has been proved that plasma APOC3 concentrations are increased in diabetic patients and are associated with a higher chance of atherosclerosis and CVD risk [82]. Apoc3 increases TG levels through mechanisms that are presented in Figure 6 [91].

Currently, there can be distinguished three main apoC3 inhibitors: volanesorsen, olezarsen, and ARO-APOC3. Volanesorsen (ISIS 304801; ISIS-ApoC-III Rx) is a second generation 2′-O-methoxyethyl (2′-MOE) antisense oligonucleotide (ASO) [91,92]. It is administered subcutaneously and, by the inhibition of APOC3 mRNA, it blocks the synthesis of the apoC3 protein in the liver. Olezarsen (ISIS 678354; AKCEA-APOCIII-LRx) is an N-acetyl-galactosamine (GalNAc)-conjugated ASO that specifically targets hepatic APOC3 mRNA to also block apoC3 protein production. There is a large quantity of Gal-NAc receptors on the hepatocytes, which increases binding capacity and affinity. It may allow for the lessening of the dosage of drugs and decrease the risk of possible toxicity [91,93]. ARO-APOC3 administration has been focused on patients with severe HTG and pancreatitis risk [91]. A clinical trial was performed that examined the influence of volanesorsen on hypertriglyceridemia with FCS [94]. It was a phase 3 trial, randomized and double-blind, that lasted 52 weeks and assessed the safety and effectiveness of volanesorsen on 66 participants with FCS. The participants were randomly assigned in a 1:1 ratio; the first group was given volanesorsen, and the second group was given a placebo. As for the results, the patients who received volanesorsen had a decrease in apo C3 levels in mean plasma—an 84% decrease at 3 months compared to the beginning and a 77% decrease in mean triglyceride levels. The patients who received the placebo had an increase in apo C3 levels in mean plasma—6.1% compared to the beginning and an 18% increase in mean triglyceride levels. There were two most frequent adverse events that appeared among patients who received volanesorsen: local injection site reactions and a decreased platelet count [94,95].

### 5.7. Lomitapide

Lomitapide is a novel lipid-lowering drug with a distinct mode of action that is not reliant on LDL-receptors. It operates by inhibiting the function of the microsomal triglyceride-transfer protein (MTP).

The phase 3 trial showed that adding lomitapide to current LLTs reduced LDL-C levels by 50% from baseline at week 26 (*p* < 0.0001). What is more, scientists observed decreased levels of TC, apoB, and TGs. After 26 weeks of the trial, the patients continued their treatment with lomitapide until week 78 for the purpose of safety evaluation. The majority of observed adverse events were related to gastrointestinal symptoms [96]. Furthermore, the report of the Italian subgroup of the clinical trial showed similarities in reducing LDL-C levels and tolerating lomitapide in the Italian cohort compared to the entire study population [97].

The Lomitapide Observational Worldwide Evaluation Registry (LOWER) study enrolled 187 patients from different countries, who, within 3 years, were treated with lomitapide. Out of these, 111 patients were in the registry, with 67 patients treated with lomitapide, and 44 were in the registry but were not treated with lomitapide. The efficacy of lomitapide treatment was noticeable across the study in patients who persisted on lomitapide. In 58.4% of the patients, a reduction in LDL-C of at least 50% from baseline was observed. The greater part of the patients treated with lomitapide (140 patients) experienced at least one adverse event (AE), in which the most common was diarrhea. Additionally, 41 patients had serious adverse events (SAEs). Cardiac disorders and infections were the most common SAEs. The study also noted that AEs were less severe than in the phase III trial, which was potentially associated with a lower dose of lomitapide [98].

In real-world experience, patients with a history of treatment for at least 6 months with lomitapide in addition to LLTs were collected retrospectively to include in the analysis. Lomitapide was prescribed at dosages between 5 and 60 mg/dL. A substantial decrease in both TG and non-HDL-C levels was observed, indicating a significant reduction (*p* < 0.0001). Additionally, there was a non-significant increase in HDL-C levels during the follow-up. Moreover, taking the highest dosage of lomitapide was associated with the lowest mean LDL-C levels. Throughout the follow-up, not a single patient had to discontinue lomitapide treatment because of any liver or gastrointestinal AEs [99].

The study of an Italian cohort treated with lomitapide and a French cohort treated with LA (lipid apheresis) suggests that lomitapide, in addition to statin/ezetimibe therapy, could be potentially more effective than LA in achieving a sustained reduction in LDL-C levels in the long term [100].

The connection of lomitapide with standard LLTs caused a reduction in LDL-C of about 56% at 2 years. During the follow-up, the reduction in LDL-C levels was consistently maintained throughout the entire 9-year study [101]. Subanalysis showed that lomitapide is not only efficacious and safe for patients with classical HoFH but also for those with ARH [102].

In the Japanese population, average reductions in LDL-C levels of about 50% from baseline for >60 weeks were observed. The mean levels of TC, TGs, and non-HDL-C were also widely reduced by lomitapide treatment. Chest pain and upper abdominal pain were noticed as the most common AEs; also, anemia and eczema were documented. And one patient presented an abnormal liver function test [103].

Patients with HoFH are at great risk of premature ASCD. Lomitapide has significant influence in lowering LDL-C, but also may reduce or stabilize carotid intima media thickness (CIMT), which is used to diagnose the extent of carotid atherosclerotic vascular disease [104] or may reduce cardiovascular events in long-term therapy. However, more studies are required to prove the beneficial effects of lomitapide in these respects [105].

Long-term safety, in particular the hepatic function of lomitapide treatment, is the subject of research by scientists. The data analysis showed that lomitapide has been associated with temporary elevations in liver transaminases, both alanine aminotransferase (ALT) and aspartate aminotransferase (AST), with no occurrences of increases in bilirubin levels, a moderate buildup of fat in the liver, and elevations in hepatic biomarkers and hepatic stiffness. However, the analysis did not show the clinical impact of lomitapide treatment on liver damage during the long-term study [106].

The researchers focused also on the impact of lomitapide treatment on TG levels in their investigations, especially in patients with FCS.

The LOCHNES study showed that after 26 weeks follow-up, median fasting TGs were reduced by 70.5% from baseline (*p* < 0.0001). Noticeable AEs were mostly gastrointestinal problems. Transaminase levels increased > 3xULN, and hepatic fat increases of 12.0–32.5% from the median were noticed. However, the median hepatic stiffness remained within the normal range. More studies are required to establish lomitapide treatment in patients with increased TG levels and who suffer from FCS [107].

### 5.8. Cholesteryl Ester Transfer Protein (CETP) Inhibitors

A different group of medicaments that was hoped to be used in dyslipidemia treatment is cholesteryl ester transfer protein inhibitors (CETPis). The mechanism of these medications is based on decreasing the transport of cholesteryl esters from HDL-C to the apolipoprotein-like VLDL lipoprotein or LDL, while simultaneously exchanging TGs [108]. This group, among others, includes torcetrapib, anacetrapib, dalcetrapib, evacetrapib, and the latest developed one, which is obicetrapib. They are efficient in terms of reducing the concentration of both LDL-C and apoB, while currently elevating the level of HDL-C. Despite the fact of lifting HDL-C concentration, they do not show a significant effect on reducing cardiovascular risk, and some of them can even elevate CVD hazard. To exemplify, the trial of torcetrapib, the first invented CETPi, was rapidly terminated due to an increased frequency of CVD events and death rate in patients using this medicament. On the other hand, anacetrapib did not present any significant adverse effects or safety problems in the course of the trial, but it is also not in use, as well as other medications belonging to this group. Despite these facts, the mentioned medicaments can serve as initial foundations on the path leading to the development of less hazardous and more effective CETP inhibitors. Additional research on this pathway is necessary. Furthermore, CETP inhibitors are able to fulfill a different role, which is protection against the development of diabetes mellitus, by increasing insulin responsiveness and boosting glucose tolerance [109,110,111].

This protective ability can reduce the risk of new-onset diabetes mellitus by up to 16%. According to that, these medicaments also play an important role in the prevention of diabetic dyslipidemia occurrence [112]. The positive impact on glucose metabolism may be at least partially linked with an increase in the level of HDL-C [113].

## 6. Conclusions

Dyslipidemia can be described as a metabolic disturbance within the lipid profile. It may be caused by various factors, including inherited and acquired elements. Presently, an increasing number of individuals are affected by this disorder, primarily attributed to leading unhealthy lifestyles. Recent advancements in diagnostic methods, more precise laboratory and genetic tests, allow diagnosing people in the asymptomatic phase of dyslipidemia. Additionally, the development of genetic and biological research provides valuable insights into the biological mechanisms of dyslipidemia, which are the vital factors to the formation of new drugs with a strictly targeted mechanism of action. The prominent example of such a drug is alirocumab. Through its mechanism, it lowers the levels of LDL-C, non-HDL-C, and others. There are some indications for its usage, such as FH, and it demonstrates potential positive cardiovascular effects and a relatively high tolerability profile. These characteristics highlight the potential significance of alirocumab in the future treatment of dyslipidemia. Another noteworthy agent is bempedoic acid, an orally administered pro-drug that lowers LDL-C levels and exhibits evidence of good tolerability and safety. It also reduces the risk of reinfarction. It is highly possible that bempedoic acid will be included in the treatment of patients with dyslipidemia. Advanced research has demonstrated the potential of ASOs to impact mRNA and provide new avenues for the treatment of persistent disorders. Current research has focused on the reduction in Lp(a) levels, showcasing promising results. Moreover, inhibitors of ANGPTL have shown the ability to lower TG and LDL levels. It has been shown that they can be particularly helpful in cases where basic treatment is not sufficient, such as in FH. Other drugs with a unique targeted mechanism of action are APOC3 inhibitors; by their mechanism, they inhibit apoC3 protein formation and, in consequence, lower TG levels. This way, it may also decrease the risk of atherosclerosis or CVD among patients. The current results are very promising and might suggest the further development of this drug. In the future, this may allow for the more efficient treatment of diseases such as FCS. Lomitapide may also be an effective lipid-lowering drug. Due to its different mechanism of action than statins or ezetimibe, it may be added as another lipid-lowering drug in order to achieve a better therapeutic effect. This is especially important in patients with HoFH, in whom standard treatment does not always bring the intended clinical effect. However, in including lomitapide in the therapy, the crucial thing is to remember the possible side effects, in particular the increase in liver tests. Not only are the levels of LDL-C and TGs the subject of research by scientists; clinicians have also been focusing their attention on the drugs that are able to elevate HDL-C levels. The greatest example is CETPis, which have the ability to decrease LDLc and ApoB levels while increasing HDL-C concentration. The HDL-C level elevation may be partially responsible for the improvement of glucose metabolism. This further results in the reduced risk of developing new-onset diabetes mellitus, and by that, also reduces the risk of diabetic dyslipidemia in the future. Despite these facts, the medicaments from this group are not currently being used.

Previously mentioned drugs are taken in combination with the original LLTs with statins and ezetimibe. The patient must be tested for whether he has a low production of LDL receptors, a high level of LPL activity, high production of PCSK9, a high activity of MTP, and a high plasma Lp(a) concentration. It also includes genetic tests. These diagnostic tests are expensive and not generally available.

The usage of new lipid-lowering drugs discussed in our article, along with the elimination of secondary factors, promoting a healthy diet, physical exercise, and achieving weight loss, may be the fundamental aspects in attaining positive clinical outcomes and effectively controlling cholesterol levels in today’s world.

## Figures and Tables

**Table 1:** Side effects of Alirocumab.

Side Effects
nasopharyngitis
injection site reactions
influenza
urinary tract infection
diarrhoea
bronchitis
myalgia
muscle spasms
sinusitis
cough
contusion
musculoskeletal pain
hypersensitivity
nummular eczema
hypersensitivity vasculitis
elevated liver enzymes

**Table 2:** Results of phase 2 clinical trial.

Dosage and Intervals	Reduction of Lp (a)
20 mg every 4 weeks	35%
40 mg every 4 weeks	56%
20 mg every 2 weeks	58%
60 mg every 4 weeks	72%
20 mg every week	80%
Placebo	6%

## Data Availability

The data used in this article are sourced from materials mentioned in the References section.
